# Psychological symptoms in Chinese nurses may be associated with predisposition to chronic disease: a cross-sectional study of suboptimal health status

**DOI:** 10.1007/s13167-020-00225-y

**Published:** 2020-10-14

**Authors:** Jinxiu Zhu, Wenjuan Ying, Li Zhang, Gangyi Peng, Weiju Chen, Enoch Odame Anto, Xueqing Wang, Nan Lu, Shanshan Gao, Guihai Wu, Jingyi Yan, Jianfeng Ye, Shenglin Wu, Chengzhi Yu, Minghui Yue, Xiru Huang, Nuo Xu, Pengxiang Ying, Yanhong Chen, Xuerui Tan, Wei Wang

**Affiliations:** 1 Department of Cardiovascular Medicine, The First Affiliated Hospital of Shantou University Medical College, Shantou, 515041 Guangdong China; 2 Institute of Clinical Electrocardiography, The First Affiliated Hospital of Shantou University Medical College, Shantou, 515041 Guangdong China; 3 Nursing Research Institute, The First Affiliated Hospital of Shantou University Medical College, Shantou, 515041 Guangdong China; 4 Nursing Department, Foshan First People’s Hospital, Foshan, 528000 Guangdong China; 5 Division of Medical Administration, Health commission of Guangdong Province, Guangzhou, 510060 China; 6 Nursing Department, The First Affiliated Hospital, Ji’nan University, Guangzhou, 510630 China; 7 School of Medical and Health Sciences, Edith Cowan University, Perth, WA 6027 Australia; 8 Clinical Research Center, The First Affiliated Hospital of Shantou University Medical College, Shantou, 515041 Guangdong China

**Keywords:** Suboptimal health status (SHS), Predictive, preventive, and personalized medicine (PPPM), Self-Rating Depression Scale (SDS), Self-Rating Anxiety Scale (SAS), Nurse, Psychological symptoms (PS), Fatigue, Cardiovascular, Digestive, Immune, Mental, Physical exercise, Marital status, Education, Chronic disease

## Abstract

**Background:**

Suboptimal health status (SHS) is a reversible state between ideal health and illness and it can be effectively reversed by risk prediction, disease prevention, and personalized medicine under the global background of predictive, preventive, and personalized medicine (PPPM) concepts. More and more Chinese nurses have been troubled by psychological symptoms (PS). The correlation between PS and SHS is unclear in nurses. The purpose of current study is to investigate the prevalence of SHS and PS in Chinese nurses and the relationship between SHS and PS along with predisposing factors as well as to discuss the feasibility of improving health status and preventing diseases according to PPPM concepts in Chinese nurses.

**Methods:**

A cross-sectional study was conducted with the cluster sampling method among 9793 registered nurses in Foshan city, China. SHS was evaluated with the Suboptimal Health Status Questionnaire-25 (SHSQ-25). Meanwhile, the PS of depression and anxiety were evaluated with Self-Rating Depression Scale (SDS) and Self-Rating Anxiety Scale (SAS) self-assessment questionnaires. The relationship between PS and SHS in Chinese nurses was subsequently analyzed.

**Results:**

Among the 9793 participants, 6107 nurses were included in the final analysis. The prevalence of SHS in the participants was 74.21% (4532/6107) while the symptoms of depression and anxiety were 47.62% (2908/6107) and 24.59% (1502/6107) respectively. The prevalence of SHS in the participants with depression and anxiety was significantly higher than those without the symptoms of depression (83.3% vs 16.7%, *P* < 0.001) and anxiety (94.2% vs 5.8%, *P* < 0.0001). The ratio of exercise habit was significantly lower than that of non-exercise habit (68.8% vs 78.4%, *P* < 0.001) in SHS group.

**Conclusions:**

There is a high prevalence of SHS and PS in Chinese nurses. PS in Chinese nurses are associated with SHS. Physical exercise is a protective factor for SHS and PS so that the exercise should be strongly recommended as a valuable preventive measure well in the agreement with PPPM philosophy. Along with SDS and SAS, SHSQ-25 should also be highly recommended and applied as a novel predictive/preventive tool for the health measures from the perspectives of PPPM in view of susceptible population and individual screening, the predisposition to chronic disease preventing, personalization of intervention, and the ideal health state restoring.

## Introduction

The nursing is invariably recognized as one of the most stressful and demanding occupation. Nurses regularly experience a variety of work-related stressors including but not limited to long work hours, shift, patient complaints and low income. With such demanding occupations could have a severe impact mental health and quality of life, including depression symptoms and anxiety symptoms as well as physical effects, such as pain, fatigue, decreased immunity, and so on [1]. Therefore, it is very important to pay attention to the psychological and physical health of nurses and the relationship between them.

Suboptimal health status (SHS) has been recognized globally as a health problem among African, Asian and Caucasian populations [2–7]. It is a reversible borderline state between health and disease, characterized by declines in vitality, physiological function, and the capacity for adaptation, and does not meet the diagnostic criteria of any medical conditions but reflects the subclinical and reversible stage of a chronic condition, characterized by the perception of health complaints, general weakness and low energy [8]. Suboptimal Health Status Questionnaire-25 (SHSQ-25) consists of 25 components related to 5 domains of health measures: fatigue, cardiovascular system, digestive tract, immune system and mental state [9, 10]. SHSQ-25 has been proved to be a reliable and valid health measure tool in large scale health status surveys in different ethnic groups [7, 11, 12].

Accumulating evidences suggests that SHS is associated with mental health disorders [13], psychosocial stress [14], cognitive disorders [15]. Psychosocial or psychological factor(s) is defined as one of the four categories of fundamental factors influencing health, others including chemical, physical and biological factors [16]. Psychological and physical health is receiving increasing attention. Recent epidemiological data suggested that 35.8% to 61.7% of nurses suffered from depressive symptoms [17, 18]. Depression and anxiety are considered the most frequent mental health disorders and they are associated with severe consequences and, therefore, not only affect the quality of human life and social function, but also contribute to the growth of social problems and economic losses [19–21].

The evolving medical model is changing from delayed to predictive, preventive and personalized medicine (PPPM). It is applicable not only to chronic and non-communicable diseases but also to predictive models, preventive measures, and personalization of medical services in case of infectious diseases and global epidemics such as COVID-19 [22].

The purpose of current study is to investigate the prevalence of SHS, PS such as depression and anxiety symptoms by means of a cross-sectional study in Chinese nurses and the relationship between SHS and PS along with predisposing factors as well as to discuss the feasibility of improving health status and preventing diseases according to PPPM concepts in Chinese nurses.

## Participants and methods

### Ethics statement

The study was approved by the First Affiliated Hospital of Shantou University Medical College Ethics committee (No. 2018100). Chinese Clinical Trial Registry was completed prior to the starting of the study (No. ChiCTR1800020214). The written informed consent was obtained from all participants prior to their inclusion in the study.

### Inclusion criteria

The inclusion criteria were (1) no history of somatic diseases, (2) no history of psychological illnesses, (3) no psychiatric abnormalities currently, (4) no history of medication consumption in the previous 2 weeks, and (5) registered in-service nurses.

### Exclusion criteria

The exclusion criteria were (1) clinically diagnosed diseases, (2) unable to complete the questionnaires, and (3) refusal to provide informed consent.

### SHS, depression, and anxiety assessments

A general questionnaire was applied to investigate demographic information and history of diseases. Questionnaires of SHSQ-25, SDS and SAS were used to evaluate SHS, depressive symptoms and anxiety symptoms, respectively. Each item was displayed as a specific statement based on how often they underwent uncomfortable symptoms in the preceding periods. Particularly, the individual’s health condition was assessed based on whether they suffered any suboptimal health symptoms during the last 3 months whereas both depressive symptoms and anxiety symptoms were assessed based on whether they suffered such symptoms during the preceding 1 week. According to the description of each item in each questionnaire, the participants answered the questions in a cell phone or with a computer within 60 min. Each item was scored depending upon the scoring rules of each questionnaire, and then the total score of each questionnaire was calculated by summing the listed points. Based on SHSQ-25, the health status of participants was stratified into two groups, the ideal health with summed SHS score < 35 and the suboptimal health with summed SHS score ≥ 35. The higher the SHS score, accompanied the worse the health status [23]. The serious degree of depressive symptoms and anxiety symptoms was estimated depending upon the scores of SDS and SAS receptively [24, 25]. The total score of less than 50 was considered normal; equal or over 50 of the total score was recognized exhibiting depressive symptoms and anxiety symptoms. The higher the score, the more serious the condition.

### Participants’ recruitment

A cross-sectional study with the cluster sampling method was conducted at hospitals and health centers, Foshan city, China, from September 2018 to January 2019. Nine thousand seven hundred ninety-three professionally registered nurses in the region, aged between 18 years old and 60 years old, were preliminarily recruited and 7959 of them completed the questionnaire survey. The participation rate was 81.27% (7959/9793). Unqualified cases who were diagnosed as hypertension (294), diabetes (268), previous medical history (447) and those with unintelligible data (843) were excluded. A total 6107 participants with women dominating (5890/6107, 96.4%) met the criteria were finally enrolled in the study.

### Grouping and statistical analysis

The statistical analysis for the data was performed via SPSS (version 24.0, IBM, New York, USA). Quantitative data normally distributed were expressed as means and standard deviation (mean *±* SD) while non-normally distributed data were expressed as quartiles (*P25~P75*). A Mann-Whitney test or a Kruskal-Wallis test was used to compare the differences between groups. Qualitative data were presented as a percentage or composition ratio. A Pearson chi-squared (*χ*^2^) test was used to compare the differences between groups. Logistic regression analysis was used for multivariate analysis with SHSQ-25 scores as the dependent variable, by which the Exp(B) and its 95% confidence intervals (CI) were obtained. *P* < 0.05 was considered statistically significant.

For comparison of SDS or SAS scores, the participants were grouped by quartile method based on SHSQ-25 scores, into 4 groups in both sexes as follows: Group *A*) SHSQ-25 score ≤ 35, Group *B*) SHSQ-25 score 36–49, Group *C*) SHSQ-25 score 50–59 and Group *D*), SHSQ-25 score ≥ 60. *F-test* and *Q-test* were used to identify the significance. The SDS or SAS score in each group was expressed as histogram (mean ± SE).

According to the diagnostic cutoff value “50” for SDS or SAS, participants were divided into two groups: abnormal groups of SDS ≥ 50 or SAS ≥ 50 and normal groups of SDS < 50 or SAS < 50. Each group was furtherly stratified according to the scores obtained from each of the five domains of the SHSQ-25: mental status, immune system, digestive tract, cardiovascular health and fatigue. The differences between the 5 domains of SHSQ-25 and the normal and abnormal SDS or SAS groups were measured by *t* test (normal distribution) or adjusted *t* test (non-Gaussian distribution).

For the analysis of the influence of exercise intervention on SHS, and symptoms of depression and anxiety, the participants were divided into four subgroups depending upon their weekly exercise frequency (never = *α*, once a week = *β*, 2–3 times a week = *γ*, and over 3 times a week = *δ*). The SDS scores, SAS scores, and SHSQ-25 scores of their four subgroups were compared by using *ANOVA* and *Q-test*.

## Results

Of 6107 eligible participants, 74.2% (4532/6107) had SHS whereas 25.8% (1575/6107) had optimal health (OPH). In terms of SHS, the same as OPH, there were significant differences on aging (*P* = 0.001), gender (*P* < 0.001), marital status (*P* = 0.013), level of education (*P* = 0.006), exercise status (*P* < 0.001), monthly income (*P* = 0.002), and academic title (*P* < 0.001), whereas no differences on smoking status (*P* = 0.388), drinking behavior (*P* = 0.325), and shift work (*P* = 0.246). There were statistically significant differences in the average SBP (*P* = 0.004) and heart rate (*P* = 0.004) between the SHS between OPH registered nurse. Conversely, a non-statistically significant difference in mean DBP (*P* = 0.077) and BMI (*P* = 0.360) was observed between the two groups (Table 1).

**Table 1:** Comparison of general characteristics between OPH and SHS groups

Variables	*n*	OPH *n* = 1575 (25.8%)	SHS *n* = 4532 (74.2%)	*t/χ*^2^	*P*
Age (years)^‡^				24..237	< 0.001
18–29	2998	856 (28.6)	2142 (71.4)		
30–39	2017	475 (23.5)	1542 (76.5)		
40–49	938	211 (22.5)	727 (77.5)		
50–60	154	33 (21.4)	121 (78.6)		
Gender^‡^				36.117	< 0.001
Male	217	94 (43.3)	123 (56.7)		
Female	5890	1481 (25.1)	4409 (74.9)		
SBP (mmHg)^†^	6107	112.21 ± 10.42*	111.32 ± 10.56*	2.869	0.004
DBP (mmHg)	6107	66.66 ± 5.75*	66.32 ± 6.10*	1.769	0.077
Heart rate (bpm)^†^	6107	74.01 ± 10.57*	75.21 ± 11.52*	− 2.895	0.004
BMI	6107	21.01 ± 2.86*	21.09 ± 2.87*	− 0.916	0.360
Marital status^†^				10.839	0.013
Single	2243	626 (27.9)	1617 (72.1)		
Married	3749	927 (24.7)	2822 (75.3)		
Divorce	91	19 (20.9)	72 (79.1)		
Death of a spouse	24	3 (12.5)	21 (87.5)		
Education^†^				12.555	0.006
Polytechnic school	310	83 (26.8)	227 (73.2)		
Junior college	2755	767 (27.8)	1988 (72.2)		
University graduate	2986	710 (23.8)	2276 (76.2)		
Master or above	56	15 (26.8)	41 (73.2)		
Smoking status				0.746	0.388
Yes	104	23 (22.1)	81 (77.9)		
No	6003	1552 (25.9)	4451 (74.1)		
Drinking behavior				0.968	0.325
Yes	263	61 (23.2)	202 (76.8)		
No	5844	1514 (25.9)	4330 (74.1)		
Exercise^‡^				74.898	< 0.001
Yes	2578	811 (31.5)	1767 (68.8)		
No	3529	764 (21.6)	2765 (78.4)		
Shift work/month				2.801	0.246
≤ 1	1017	281 (27.6)	736 (72.4)		
2–4	1532	378 (24.7)	1154 (75.3)		
≥ 5	3558	916 (25.7)	2642 (74.3)		
Monthly income (RMB)^†^				16.820	0.002
< 2000	94	32 (34.0)	62 (66.0)		
2000–4000	1756	494 (28.1)	1262 (71.9)		
4001–6000	2007	528 (26.3)	1479 (73.7)		
6001–8000	1415	328 (23.2)	1087 (76.8)		
> 8000	835	193 (23.1)	642 (76.9)		
Academic title^‡^				28.543	< 0.001
Assistant—nurse	185	62 (33.5)	123 (66.5)		
Primary	4360	1160 (26.61)	3200 (73.39)		
Intermediate	1374	307 (22.3)	1067 (77.7)		
Advanced	188	46 (24.47)	142 (75.53)		

*OPH*, optimal health; *SBP*, systolic blood pressure; *DBP*, diastolic blood pressure; *BMI*, body mass index; *bpm*, beats per minute; *RMB*, Renminbi

1 RMB ≈ 0.125 EUR and 1 EUR ≈ 8.011 RMB; 1 RMB ≈ 0.148 USD and 1 USD ≈ 6.77 RMB (exchange rate on 19 September 2020); shift work was defined as at least 1 night shift per month, and the night shift mode is 8 h of continuous work outside 8 am–6 pm, grouped by quartile

*Average values

†*P* < 0.05

‡*P* < 0.001

Of 6107 eligible participants, 74.2% (4532/6107) had SHS whereas 25.8% (1575/6107) had optimal health (OPH). In terms of SHS, the same as OPH, there were significant differences on aging (*P* = 0.001), gender (*P* < 0.001), marital status (*P* = 0.013), level of education (*P* = 0.006), exercise status (*P* < 0.001), monthly income (*P* = 0.002) and academic title (*P* < 0.001), whereas no differences on smoking status (*P* = 0.388), drinking behavior (*P* = 0.325) and shift work (*P* = 0.246). There were statistically significant differences in the average SBP (*P* = 0.004) and heart rate (*P* = 0.004) between the SHS between OPH registered nurse. Conversely, a non-statistically significant difference in mean DBP (*P* = 0.077) and BMI (*P* = 0.360) was observed between the two groups

SHS registered nurses had significantly higher mean SDS score (50.69 versus 44.47, *P* < 0.001) and SAS (45.03 versus 36.50, *P* < 0.001) compared with OPH registered nurses. A significantly higher proportion of the population beyond the threshold value ≥ 50 for SDS score (83.3% versus 66.0%, *P* < 0.001) and the population beyond the threshold value ≥ 50 for SAS score (94.2% versus 67.7%, *P* < 0.001) than those with the values < 50 (Table 2).

**Table 2:** Comparison of SDS, SAS, and basic characteristics between OPH and SHS groups

Variables	*n*	OPH *n* = 1575 (%)	SHS *n* = 4532 (%)	*t*/*χ*^2^	*P*
SDS^‡^	6107	44.47 ± 10.50*	50.69 ± 10.80*	− 19.808	< 0.001
< 50	3199	1089 (34.0)	2110 (66.0)	239.019	< 0.001
≥ 50	2908	486 (16.7)	2422 (83.3)		
SAS^‡^	6107	36.50 ± 7.43*	45.03 ± 10.02*	− 35.650	< 0.001
< 50	4605	1488 (32.3)	3117 (67.7)	416.215	< 0.001
≥ 50	1502	87 (5.8)	1415 (94.2)		

*OPH*, optimal health; *SDS*, Self-Rating Depression Scale; *SAS*, Self-Rating Anxiety Scale

*Average scores

**‡***P* < 0.001

SHS registered nurses had significantly higher mean SDS score (50.69 versus 44.47, *P* < 0.001) and SAS (45.03 versus 36.50, *P* < 0.001) compared with OPH registered nurses. A significantly higher proportion of the population beyond the threshold value ≥ 50 for SDS score (83.3% versus 66.0%, *P* < 0.001) and the population beyond the threshold value ≥ 50 for SAS score (94.2% versus 67.7%, *P* < 0.001) than those proportion of the population with the these values < 50

As shown in Table 3, the multivariate logistic regression analysis with SHS score as the dependent variable showed that age (*P* = 0.018), academic title (*P* = 0.005), monthly income (*P* < 0.0001), SDS (*P* = 0.005), and SAS (*P* < 0.0001) were all increased risk factors significantly associated with SHS. Conversely, regular exercise and monthly income less than 6000-yuan RMB were significantly protective factors for SHS (all *P* < 0.05).

**Table 3:** Results of multivariate logistic regression analysis with SHSQ-25 score as the dependent variable

	*B*	SE	Wald	*P*	Exp(B) 95% CI
Male	− 0.425	0.326	1.693	0.193	0.654 (0.345–1.240)
Age^**†**^	0.029	0.012	5.600	0.018	1.029 (1.005–1.054)
Marital status			1.648	0.649	
Single	− 19.294	10,672.350	0.000	0.999	0.000-
Married	− 19.462	10,672.350	0.000	0.999	0.000-
Divorce	− 19.165	10,672.350	0.000	0.999	0.000-
SBP	− 0.012	0.006	3.740	0.053	0.988 (0.976–1.000)
DBP	0.000	0.010	0.002	0.969	1.000 (0.979–1.020)
Heart rate	0.009	0.005	2.535	0.111	1.009 (0.998–1.020)
Education			1.595	0.661	
Polytechnic school	− 1.399	1.116	1.572	0.210	0.247 (0.028–2.199)
Junior college	− 1.339	1.096	1.492	0.222	0.262 (0.031–2.246)
University graduate	− 1.354	1.096	1.528	0.216	0.258 (0.030–0.210)
Academic title^†^			12.920	0.005	
Assistant—nurse	− 0.887	0.458	3.753	0.053	0.412 (0.168–1.010)
Primary	0.087	0.382	0.052	0.820	1.091 (0.516–2.305)
Intermediate	− 0.221	0.320	0.477	0.490	0.802 (0.428–1.501)
Monthly income (RMB)‡			21.826	0.000	
< 2000	− 1.351	0.455	8.819	0.003	0.259 (0.106–0.632)
2000–4000	− 0.902	0.257	12.328	0.000	0.406 (0.245–0.671)
4001–6000	− 0.777	0.247	9.875	0.002	0.460 (0.283–0.747)
6001–8000	− 0.224	0.242	0.858	0.354	0.799 (0.497–1.284)
BMI	− 0.010	0.022	0.192	0.661	0.990 (0.949–1.034)
Smoking	0.718	0.485	2.189	0.139	2.051 (0.792–5.312)
Drinking	0.440	0.326	1.819	0.177	1.552 (0.819–2.940)
Exercise†	− 0.332	0.117	8.135	0.004	0.717 (0.571–0.901)
SDS^†^	0.019	0.007	8.014	0.005	1.019 (1.006–1.032)
SAS^‡^	0.117	0.009	164.601	0.000	1.125 (1.105–1.145)

*BMI*, body mass index; *SDS*, Self-Rating Depression Scale; *SAS*, Self-Rating Anxiety Scale

†*P* < 0.05

‡*P* < 0.001

1 RMB ≈ 0.125 EUR and 1 EUR ≈ 8.011 RMB; 1 RMB ≈ 0.148 USD and 1 USD ≈ 6.77 RMB (exchange rate on 19 September 2020)

The multivariate logistic regression analysis with SHS score as the dependent variable showed that age (*P* = 0.018), academic title (*P* = 0.005), monthly income (*P* < 0.0001), SDS (*P* = 0.005), and SAS (*P* < 0.0001) were all increased risk factors significantly associated with SHS. Conversely, regular exercise and monthly income less than 6000 yuan RMB were significantly (all *P* < 0.05) protective factors for SHS

**Fig. 1:**
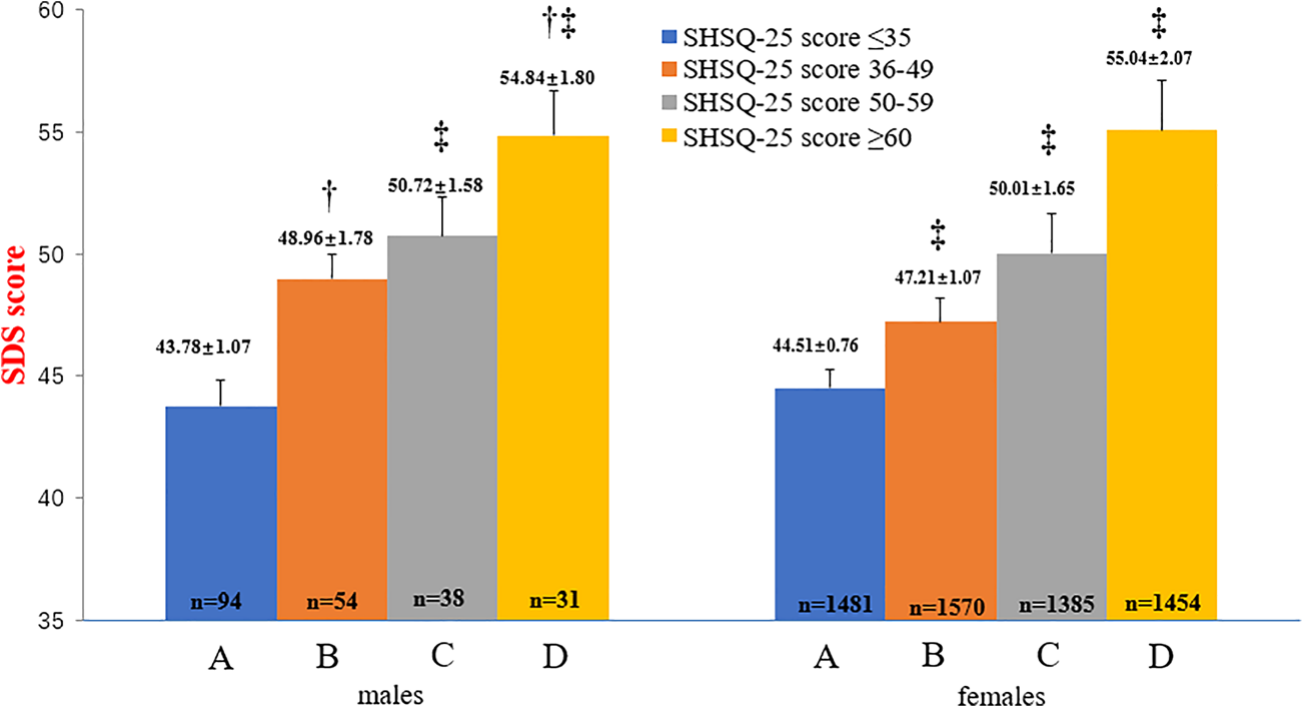
Comparison of SDS scores of male and female grouped by SHSQ-25 quartile. In the male participants, †*P* < 0.05, group B and group D. ‡ *P* < 0.001, group A and group C, group A and group D. In the female participants, ‡*P* < 0.001, from group A to group D, between any two groups. The results indicated that SHSQ-25 score (depending on quartile scores) increases with SDS score increases in both male and female registered nurses

The average SDS score increased with significantly increasing SHS quartile scores among both male and female registered nurses. Compared with the male registered nurses who were in the first quartile of SHS score (≤ 35), there were statistically significant differences between the average SDS score among males in the second quartile (36–49) (*P* = 0.006), third quartile (50–59) (*P* < 0.001) and fourth quartile (≥ 60) (*P* < 0.001). In addition, there were significant differences between the average SDS score between male registered nurses in the second SHS quartile (36–49) compared with those in the fourth quartile (≥ 60) (*P* = 0.018). Among the female registered nurses, there were significant differences in the average SDS scores between all four quartiles of the SHS scores (all pairs *P* < 0.001) (Fig. 1).

**Fig. 2:**
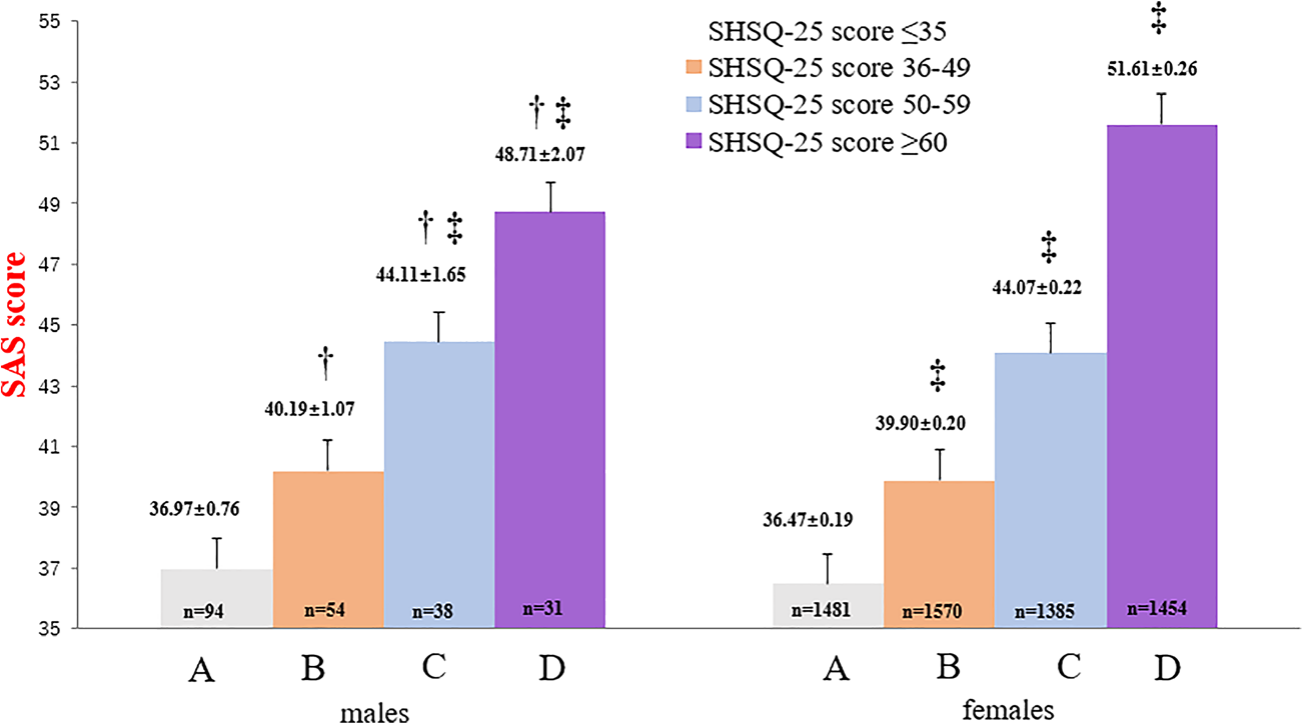
Comparison of SAS scores of men and women grouped by SHSQ-25 quartile. In the male participants, †*P* < 0.05, group A and group B, group B and group C, and group C and group D; ‡ *P* < 0.001, as compared with group A and group C, group A and group D. In the female participants, ‡*P* < 0.001, from group A to group D, between any two groups. The results indicated that SHSQ-25 score (depending on quartile scores) increases with SAS score increases in both male and female registered nurses

The average SAS score increased significantly with the increasing quartile scores of SHS among both male and female registered nurses (Fig. 2). Compared with the male registered nurses who were in the first quartile of SHS score (≤ 35), there were statistically significant differences between the average SAS score among males in the second quartile (36–49) (*P* = 0.032), third quartile (50–59) (*P* < 0.001) and fourth quartile (≥ 60) (*P* < 0.001). In addition, there were significant differences between the average SAS score between male registered nurses in the second SHS quartile (36–49) compared with those in the third quartile (50–59) (*P* = 0.035), and between males in the third SHS quartile (50–59) compared with those in the fourth quartile (≥ 60) (*P* = 0.035). Among the female registered nurses, there were significant differences in the average SAS scores between all four SHS quartile scores (all pairs *P* < 0.001) (Fig. 2).

**Fig. 3:**
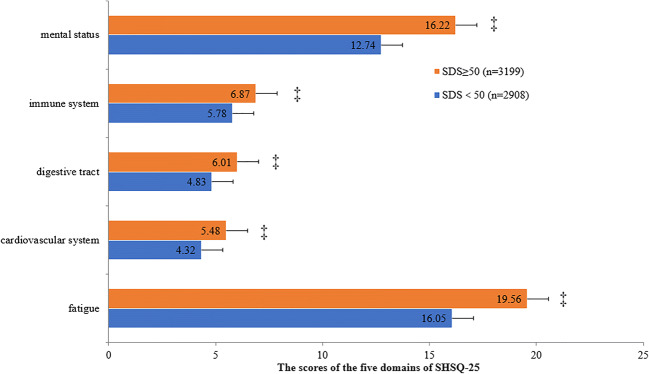
Comparison of the scores of the five domains of SHSQ-25 between the normal and abnormal scoring groups depending on SDS scores. ‡*P* < 0.001. The results showed that the scores of participants of SDS ≥ 50 were significantly higher than those of SDS < 50 no matter which one of the five domains of SHSQ-25

The average SHS specific domains scores of mental health status, immune system, digestive tract, cardiovascular health and fatigue were statistically significantly increased among the registered nurses who had SDS score ≥ 50 compared with those with SDS score < 50 (all *P* < 0.001) (Fig. 3).

**Fig. 4:**
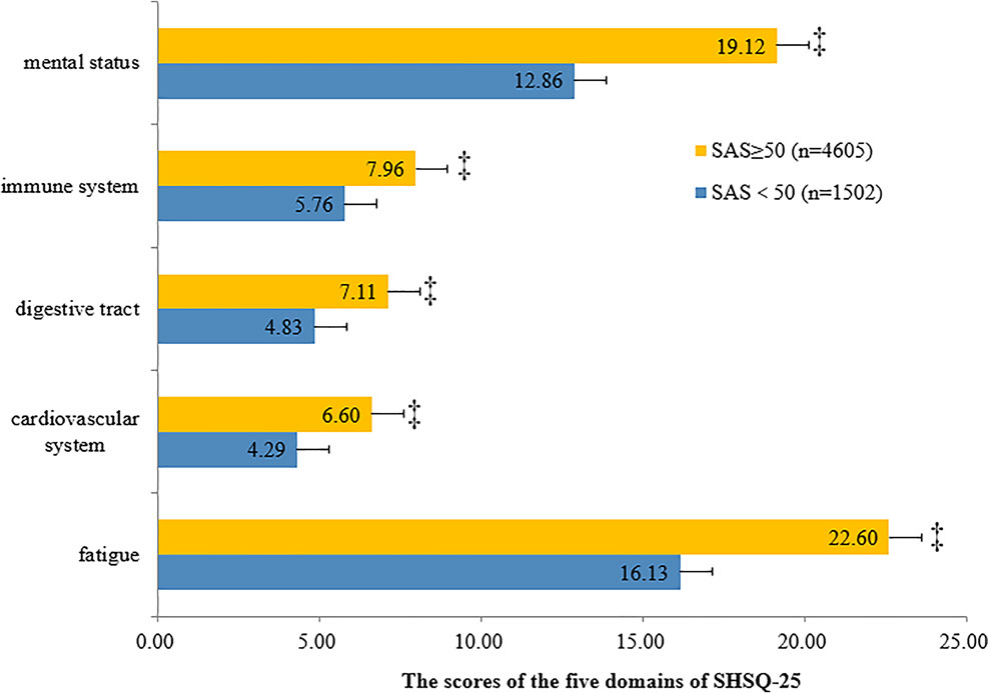
Comparison of the scores of the five domains of SHSQ-25 between the normal and abnormal scoring groups depending on SAS scores. ‡*P* < 0.001. The results showed that the scores of participants of SAS ≥ 50 were significantly higher than those of SDS < 50 no matter which one of the five domains of SHSQ-25

Similarly, the average SHS specific domains scores of mental health status, immune system, digestive tract, cardiovascular health and fatigue were statistically significantly increased among the registered nurses who had SAS score ≥ 50 compared with those with SDS score < 50 (all *P* < 0.001) (Fig. 4).

**Fig. 5:**
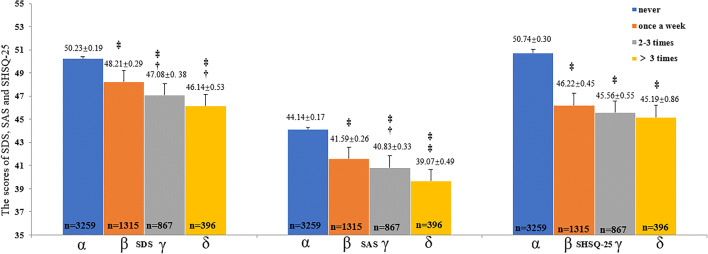
The influence of exercise on SHSQ-25, SDS and SAS. †*P* < 0.05; ‡*P* < 0.001. The participants were divided into four subgroups depending upon their exercise frequency each week (never = *α*, once a week = *β*, 2–3 times a week = *γ*, and over 3 times a week = *δ*). The SDS scores, SAS scores, and SHSQ-25 scores of the four subgroups were compared by using ANOVA and *Q*-test. The results showed that the scores of SHSQ-25, SDS, and SAS decreased with the increase of exercise frequency, suggesting that the exercise is a protective factor for SHS and PS

As shown in Fig. 5, the frequency of regular exercise had a significant inverse association with the overall SHS score, and PS assessed by the SDS and SAS. Specifically, compared with the registered nurses who never undertook physical exercise, those who did exercise at least once per week, 2–3 times per week and more than 3 times per week had significantly reduced average SHS, SDS and SAS scores (all *P* < 0.001). Overall, the frequency or increasing rate of regular exercise per week was significantly associated with a more reduced average SHS, SDS, and SAS scores.

## Discussion

### High prevalence of SHS in Chinese nurses

In China, as the pace of work and lifestyle accelerate, and the environment and pollution deteriorate, the prevalence of SHS continues to rise, with 17.8–60.5% of people currently reported to have SHS [26, 27]. SHS are associated with cardiovascular disease, diabetes and other chronic diseases [28, 29].

Previous studies showed that the respective prevalence of SHS in Chinese college students is 21.0%, medical students 25.4%, and non-medical students 14.5% [23]. A geographical-based investigation showed the SHS prevalence of 46.3% among 24,475 individuals aged 15–60 years from South China [30]. The current study was also from South China and showed the SHS prevalence of 74.2% in nurses (Table 1), which was higher than that of all other populations [23, 31]. In addition, the current study showed that SHS prevalence was higher in females (74.9%) than that in males (56.7%); having a rising trend with the increase of age, i.e., aging was an independent risk factor of SHS (Table 3). Sociodemographic factors including education background, education level, economic situation and marital status among nurses were significantly associated with SHS.

### Prevalence of PS in Chinese nurses is associated with SHS, the predisposition to chronic disease

Medical workers, especially nurses, are often associated with mental health issues such as depression and anxiety [32]. Studies have shown that about 38%–61.7% of nursing staff have depressive symptoms [18, 33]. Nurses play an important key role in healthcare but work in a highly stressful and unfriendly environment. The work situation, overload works, safety factors and circadian shift model affect the physical and mental health of nursing staff in China, resulting in poor health and also a large proportion of nursing staff leaving the working force [11, 34–37]. For example, if the symptoms of depression and anxiety are not well managed, they will increase the risk of suffering cardiovascular diseases [38]. It is therefore important to understand if the symptoms of depression and anxiety are associated with poor health lacking, however, a diagnosable condition.

Depression and anxiety symptoms are the key PS that underpin several chronic diseases among health workers. A multicenter study from 13 hospitals reported that the health anxiety prevalence was 30.14% among Chinese medical workers [39]. In the present study, the prevalence of depressive symptoms and anxiety symptoms are 47.62% and 24.59%, respectively, which is consistent with those reported by other studies [39, 40]. The results only as “symptoms” did not represent a clinical diagnosis or were understood as reversible pre-depression or pre-anxiety stages. Psychological stress is a type of negative emotional state. Chronic social psychological stress caused by long-term psychological stress may lead to SHS by the changed profiling of serum cortisol level and glucocorticoid receptor [37, 41].

The present study showed that compared with the nurses without PS, the prevalence of SHS in nurses with symptoms of depression and anxiety were 83.3% and 94.2%, respectively (Table 2), which are extremely high. The results suggested that PS is important contributing factor of SHS. As the SHS score increased, the nurses were 1.02 times and 1.13 times increased risk of developing depression and anxiety symptoms, respectively (Table 3). We suspected that the negative effects of this mental state in nurses may owe to the excessive workload and worries about medical errors and accidents in their routine clinical practices. Nurses may also experience PS due to the acts of hostility and violence from patients during working hours [42].

Another finding was that as the SHS score increased, both the SDS and SAS scores increase in the same direction, with similar trends among both male and female nurses (Figs. 1 and 2). In addition, the SHS specific-domain scores were significantly higher in the groups of nurses who had high SDS and SAS scores, suggesting a direct association between SHS and psychological states in Chinese nurses. The explanation to the observed finding may owe to the shared common characteristics between SHS, depression and anxiety states, particularly the relevant items in the questionnaires and the common predisposing factors associated with their causes. The current study indicates that nurses with psychological problems are more likely to have SHS. The coexistence of SHS and psychological problems will aggravate in terms of worse life quality, higher health risks, lower work efficiency and less happiness.

Unlike the previous studies among Chinese college students and freshmen [23, 43], the present study did not find a significant relationship between SHS and smoking, alcoholic beverage intake and BMI. This may be due to the characteristics of the targeted cohort who are medical workers and most of them are women, and fewer of them had the habit of smoking and drinking. In addition, the BMI of the nurse was generally within normal values (Table 1).

It was reported previously that shift working model interferes with cortisol and melatonin rhythms, which cause negative effects on health symptoms [44]. However, this phenomenon was not observed in the present study. Whether shift working model is related to SHS or not needs further investigation.

### Blood pressure and heart rate showed significant differences between SHS and optimal health groups

Our study showed that the blood pressure and heart rate showed significant differences between SHS and OPH groups, however, both them were within the normal blood pressure ranges. Conversely, in this study there were no differences in BMI, shift working model, smoking and drinking habits between SHS and OPH groups.

### Physical exercise may be a feasible direction of intervention

In the OPH group, the proportion of physical exercise was higher than that of non-exercise ones. However, in the SHS group, the proportion of those with no physical exercise was significantly higher than those who did exercise regularly. The results of multivariate logistic regression analysis with SHSQ-25 scores as the dependent variable also showed that the exercise is a significant protective factor (*P* < 0.004) for SHS (Table 3). These results indicate that regular and adequate physical exercise is beneficial to both mental and physical health status of nursing practitioners.

A study showed that a positive lifestyle, such as regular physical exercise can reduce psychological stress and maintain optimal physical and psychological health [45]. The finding indicating the protective effect of exercise for the health status among nurses is consistent with previous studies that found the benefit of physical exercise among their study population [46, 47]. The current study also showed that the improvement effect of exercise on PS is associated and also enhanced with the frequency of exercise (Fig. 5), but we did not observe the same outcome as far as SHS was concerned; exercise was a decisive factor, but the frequency of exercise showed no significant effect on SHS (Fig. 5).

### Unique niche of PPPM and its optimal application

The PPPM proposes, implements, and supports the need to change from “reactive” to “predictive, preventive, and personalized medicine” concepts of health. It is a really complex all-encompassing approach combining advantages of other attitudes towards health and healthcare such as traditional, complementary, and alternative medicine (TCAM); person-centered medicine (PCM); individualized medicine (IM); stratified medicine (SM); personalized medicine (PM); and precision medicine (PrecMed), and minimizing their specific disadvantages. It is the clear concepts demonstrating the highest level of maturity, the most optimal strategies considering interests of healthy individuals, subpopulations, patient cohorts, health care systems and society as whole [48].

The optimal application of PPPM and the unique niche in healthcare includes, but is not limited to the predictive medicine, the new spectrum of screening programs, targeted prevention, currently unmet needs of healthy, subpopulations and patient cohorts, cost-effective medical services and optimized health care economy, new dimension of professional interests, new scale of the knowledge integration, highly motivated technological innovation, highly motivated interdisciplinary and multidisciplinary cooperation, individualized patient profiling, active participation of patients in the health care process [48–50].

The innovative PPPM is emerging as the hotspot of efforts in healthcare aimed at curbing the prevalence of chronic diseases such as cardiovascular diseases, diabetes and cancer [28, 51, 52]. PPPM concepts have actually been practically applied to cardiovascular diseases, diabetes, oral medicine, chronic diseases, as well as tumor therapy and tumor postoperative evaluation [2, 53–55]. But the study of SHS and PS related factors among nurses in English literature was not searched. Although the poor mental and body health status of nurses has less immediate impact on their daily life and work, it is a long-term accumulation of intangible occupational hazards, and impacts on the overall health of individuals [56]. On the other hand, good health condition is beneficial to make no mistakes in routine clinical practice, because any mistakes in this area can cause dissatisfaction of patients and even affect the prognosis of patients.

The SHS concept appears as one of the innovative solutions in the context of PPPM [9, 57]. Targeted prevention and then personalized treatment are necessary to delay or block the progress of the SHS into the disease state in nurses. According to the PPPM model, screening the SHS population, making disease risk prediction and then developing personalized intervention provides a window opportunity to prevent or delay the occurrence and development of a series of diseases, which has a great benefit to the existing medical model [58].

Therefore, to clarify whether the application of PPPM concepts will provide an opportunity for early intervention in SHS population screened from Chinese nurses. We will work out health interventions and then carry them out tailored to this population with the main goal of preventing “suboptimal health” from developing into chronic non-communicable diseases (NCD), such as mental health, cardiovascular disease (CVD), immune disease, digestive system disease, and cancer [58].

### Identification of PS associated with SHS from the perspective of PPPM

Health workers including nurses are mostly associated with risk of cardiovascular diseases due to unrecognized depression and anxiety symptoms, which are caused by the highly stressful and unfriendly working environment [18, 32, 33]. It is necessary to identify early indicators of poor health associated with undiagnosed depression and anxiety symptoms to enable targeted preventive measures for this group.

In terms of SHS-related issues involved in psychological factor, it is one of four categories of fundamental factors which are psychosocial or psychological, chemical, physical and biological factors influencing health [16]. The study is better coupled to the PPPM, providing a new approach that to be able to assess the health state in a complex way, we have to take into consideration these factors. It is the contribution of this study, how, actually, can such an assessment be done via using well prepared SHSQ-25, SAS and SDS questionnaires, which may become a valuable tool for practical PPPM, complementary, and cost-effective to the measuring of particular levels of selected biomarkers in the biological fluids. The most important is that we suggest what to do to prevent health deterioration, especially from a psychological point of view.

The PPPM concepts as explained by SHS are likely to support the early prediction of PS which can generate personalized interventions to achieve disease prevention. Early identification of psychological coupled with personalized intervention would prevent the progression of depression state and anxiety state progress into psychological disorders complicated with cardiovascular diseases and tumors. Therefore, it is necessary to evaluate the psychological status of nurses, especially among the elderly nurses.

Our study suggested that PPPM concepts are urgently needed for early assessment, early prevention, multidisciplinary cross-treatment and pre-disease intervention for the nurses with symptoms of depression and anxiety.

## Limitations

The results were drawn from a cohort of Chinese nurse thus these results cannot be simply generalized in the other general populations. In addition, there are no nurses who only work night shift in our study, and all nurses are shift mode, which may not fully reflect the effect of night shift on SHS. The results were drawn from a cohort of Chinese nurse thus these results cannot be simply generalized in the other general populations. In addition, there are no nurses who only work night shift in our study, and all nurses are in shift mode, which may not fully reflect the effect of night shift on SHS. Also, we expect that analogical studies can be done with different groups of nurses around the world with, of course, particularly regional deviations from our observations. Further, there was a lack of detailed parameters involved in physical exercise, such as excess post-exercise oxygen consumption (EPOC), VO_2_Max, breath rate, mileage, or similar information. These parameters should be reflected in further studies in view of the important relationship between physical exercise and PS and SHS.

## Conclusions and expert recommendations

There is a high prevalence of SHS and PS in Chinese nurses. PS in Chinese nurses is associated with SHS, which is influenced by age, monthly income and exercise habits. Physical exercise is a protective factor for SHS and PS so that the exercise should be strongly recommended as a valuable measure following the PPPM principles in this population. Besides the psychological and physical health benefits of physical exercise itself, it also reminds nurses that more free time is needed. PPPM concepts focus on prediction and individualized treatment. Prediction not only refers to disease prediction but also includes the prediction of population with high risk. For the special group of nurses, it is a long way to go to improve the mental and physical health. PPPM concepts may be the first step in the long march. Paying more attention to factors that influence SHS and PS may be helpful to improve the general health condition of nurses, thereby creating an intervention opportunity from PPPM concepts.

SHS assessment by using SHSQ-25 can effectively screen the predisposition in nurses, thus identify the group and individuals in SHS. Hence, this study, which timely provides an insight into SHS assessment of nurses, is the way forward for early identification of poor health and implementation of preventable risk factors likely to culminate in future cardiovascular illnesses. Therefore, SHSQ-25 for SHS assessment is highly recommended as a novel tool for the health measures from the perspectives of PPPM in view of susceptible population and individual screening, the predisposition to chronic disease preventing, personalization of intervention, and the ideal health state restoring. We also recommend that healthy education and intervention should focus on the five SHS health specific domains, especially mental. SHS assessment is subjective and may not represent the embodied of total health, hence we recommend future evaluation of both subjective and objective biochemical measure to understand the causal effect and systemic pathologies associated with SHS-related PS among medical workers such as nurses.

PPPM is considered the “medicine of the future” which needs the paradigm change for entire spectrum of medical research and services, improved professional and general educational levels, new economic and application models for both disease and health care [48]. It provides us with a new way of thinking in the direction that prevents the progression from subclinical as known as SHS into pathologies of chronic diseases, as well as enables the prediction of individual disease predisposition.

In the present study, the SHS and PS closely related implies that the SHS though generally perceived as “subjective” is essential for identifying poor health (subclinical), suggesting SHS as a potent subjective measure from the PPPM perspective. Integration SHS assessment in routine medical checks as a pre-health screening concept for health professionals will create opportunity for self-education and promotion, responsibility, risk assessment, disease stratification, individualized management and targeted preventive measures, which are all key policies in PPPM.

It should also be recommended that further studies and confirmations especially in other countries and ethnical groups are, of course, very valuable for PPPM. We would like to encourage our colleagues in the field around the world to perform similar surveys in different countries in order to increase the evidence for practical PPPM implementation.

## Abbreviations

BMI: body mass index
bpm: beats per minute
COVID-19: coronavirus disease 2019
CVD: cardiovascular disease
DBP: diastolic blood pressure
IM: individualized medicine
NCD: non-communicable diseases
OPH: optimal health
PCM: person-centered medicine
PM: personalized medicine
PPPM: predictive, preventive, and personalized medicine
PS: psychological symptoms
PrecMed: precision medicine
SAS: Self-Rating Anxiety Scale
SBP: systolic blood pressure
SDS: Self-Rating Depression Scale
SHS: Suboptimal health status
SHSQ-25: Suboptimal Health Status Questionnaire-25
SM: stratified medicine
TCAM: traditional, complementary, and alternative medicine
